# Evaluation of the seroprevalence of influenza A(H1N1) 2009 on a university campus: a cross-sectional study

**DOI:** 10.1186/1471-2458-11-922

**Published:** 2011-12-13

**Authors:** Shira C Shafir, Kaitlin A O'Keefe, Kimberley I Shoaf

**Affiliations:** 1 Center for Public Health and Disasters, School of Public Health, University of California, Los Angeles, CA, USA; 2 Department of Epidemiology, School of Public Health, University of California, Los Angeles, CA, USA; 3 Department of Community Health Sciences, School of Public Health, University of California, Los Angeles, CA, USA

## Abstract

**Background:**

Human infection with influenza A(H1N1) 2009 was first identified in the United States on 15 April 2009 and on 11 June 2009, WHO declared that the rapidly spreading swine-origin influenza virus constituted a global pandemic. We evaluated the seroprevalence of influenza A(H1N1) 2009 virus on a large public University campus, as well as disparities in demographic, symptomatic and vaccination characteristics of participants.

**Methods:**

Using a cross-sectional study design, sera was collected from volunteers and then tested for the presence of antibodies to the virus using a ≥ 1:40 dilution cut-off by hemagglutination inhibition assay. In conjunction, participants were asked to complete a questionnaire allowing us to estimate risk factors for infection in this population, as well as distinguish artificially derived antibodies from naturally derived antibodies.

**Results:**

300 total participants were recruited and tested. 158 (52.6%) tested positive for influenza A(H1N1) 2009 via hemagglutination inhibition assay using a ≥ 1:40 dilution cut-off. 86 people (54.4%) tested positive for H1N1 but did not report experiencing symptoms during the pandemic meeting the May 2010 CDC definition of influenza-like illness. Furthermore, of those individuals who reported that they had received the H1N1 vaccine, 16% did not test positive.

**Conclusions:**

Overall, 52.7% of the total study population tested positive for influenza A(H1N1) 2009. 54.4% of those who tested positive for influenza A(H1N1) 2009 using the ≥ 1:40 dilution cut-off on the hemagglutination inhibition assay in this study population did not report experiencing symptoms during the pandemic meeting the May 2010 CDC definition of influenza-like illness. 16% of those who reported receiving the H1N1 vaccine did not test positive by HAI. We also found that vaccination coverage for H1N1 vaccine was poor among Blacks and Latinos, despite the fact that vaccine was readily available at no cost.

## Background

Human infection with influenza A(H1N1) 2009 was first identified in the United States on 15 April 2009 and on 11 June 2009, WHO declared that the rapidly spreading swine-origin influenza virus constituted a global pandemic [1]. Influenza A(H1N1) 2009 is characterized by a combination of gene segments not previously identified [2]. Within weeks of the beginning of the epidemic, public health laboratories quickly became overwhelmed with unprecedented numbers of clinical influenza specimens for testing, and the Centers for Disease Control and Prevention (CDC) quickly recommended changes in the testing strategy [3]. The CDC recommended that since uncomplicated influenza did not require a laboratory diagnosis for clinical management, the only people who required testing for influenza were: hospitalized patients with suspected influenza, patients for whom a diagnosis of influenza would have informed decisions regarding clinical care, infection control, or management of close contacts, and patients who died of an acute illness in which influenza was suspected.

According to the CDC, diagnosis of other groups was not considered a priority for a number of reasons, the foremost of which being "Once influenza activity has been documented in a community or geographic area, most patients with an uncomplicated illness consistent with influenza can be diagnosed clinically and do not require influenza testing for clinical management, including antiviral treatment decisions [4]."

While this strategy was extremely prudent with respect to management of the resources of public health laboratories and the ability to clinically manage influenza A(H1N1) 2009 cases, in the absence of serological surveys of the population it is not possible to accurately measure the critical demographic, symptomatic and vaccination characteristics of the influenza A(H1N1) 2009 virus.

After the beginning of the global pandemic, several national representative serosurveys were conducted in order to get a picture of the population immunity profile as well as to get a picture of the infection during the first wave [5-7]. Serosurveys were also conducted among a variety of targeted populations in different regions throughout the world throughout the course of the pandemic [8-13]. To our knowledge, however, our study represents the first targeted serosurvey conducted among the high risk population of college-age students on a University campus. These results should allow for evidence-based decisions during future waves and potentially during future epidemics.

## Methods

Approval for this study was obtained from Medical IRB-2 at UCLA. Written consent was obtained from all participants.

Participants were recruited for participation via convenience sampling on the UCLA campus. Anyone who fulfilled the inclusion criteria (at least 18 years of age, able to give informed consent in English and affiliated with the UCLA community) was offered enrollment in the study. Potential participants were recruited from large gatherings of UCLA students, faculty and staff in order to maximize participation in the study. Following the process of informed consent, all participants completed a comprehensive questionnaire containing questions about their basic demographic information including their date of birth, gender, race, location of residence (on/off campus), and affiliation with UCLA (undergraduate, graduate, faculty, staff or other). The questionnaire also inquired about their vaccination status (both seasonal and H1N1 vaccine) and history of flu-like symptoms including chills, cough, diarrhea, fever, headache, muscle aches, nausea, runny nose, sore throat, stuffy nose and vomiting. They were asked whether or not they sought medical care, whether or not they had been diagnosed with H1N1 and whether or not they had been hospitalized due to H1N1 infection. They were also asked whether or not they had known exposures to individuals who had been diagnosed with H1N1 infection. In order to attempt to minimize recall bias, for each question participants were specifically prompted to think about the time frame from April 2009 until the current date (May 2010). All subjects were recruited in May of 2010.

A 10 ml venous blood sample was then collected from all willing participants. Participants were free at any point to decline to give detailed information and/or biological specimens and they were free to end their participation in the study at anytime. In total, 300 participants were enrolled in the study.

Blood specimens were centrifuged and separated into plasma and buffy-coat aliquots prior to being frozen and stored in a - 20°C freezer at UCLA until they were ready for laboratory analysis at the University of Florida. Antibody responses were detected by use of a routine standard Centers for Disease Control and Prevention protocol hemagglutination antibody inhibition (HAI) analysis to detect antibodies to influenza A(H1N1) 2009 (A/Mexico/4108/09(pandemic H1N1)). The human H1N1 strain was grown in MDCK cells. Serum samples were pretreated with receptor-destroying enzymes (1 part serum to 3 parts enzyme) from *Vibrio cholerae *overnight, and then were hemadsorbed with guinea pig blood. Serial two-fold dilutions of serum were tested beginning with a 1:10 dilution and a final dilution of 1:640. Suitable control samples were included in all assays. HI titer results were reported as the reciprocal of the highest dilution of serum that inhibited virus-induced hemagglutination of a 0.5% (from guinea pigs) solution of erythrocytes [14,15]. In order to be consistent with other published papers in the field, sera were considered positive at an HI titre of 1: 40 or greater since titres greater than 1: 40 are correlated with a reduction of 50% of the risk of contracting an influenza infection [16-23]. While the authors acknowledge this is a highly sensitive threshold, it may come at the compromise of specificity.

Analyses were conducted using the SAS software (SAS, Inc., V.9.1). A cross-sectional analysis was performed using the questionnaire data obtained from the participants. Descriptive variables were examined and then we cross-tabulated variables of interest to explore any apparent associations. When analyzing symptomatic characteristics, we looked at which participants reported the aggregate of symptoms which met the May 2010 CDC definition on influenza-like illness (fever AND cough and/or sore throat). Because of small sample size, more complex statistical analyses were not performed.

## Results and discussion

A total of 300 subjects were recruited into the study. The characteristics of study participants are presented in Table 1.

**Table 1:** Distribution of demographic characteristics

Characteristics	no. (%)
**Gender**	

Male	115 (38.3)

Female	185 (61.6)

Total	**300**

**Ethnicity**	

Asian/Pacific Islander	93 (31)

Black, Non-Latino	14 (4.6)

Latino	36 (12)

Native American/Alaskan	2 (.66)

White, Non-Latino	146 (48.6)

Mixed	6 (2)

Not Specified	3 (1)

Total	**300**

**Location of Residence**	

On campus	81 (27)

Off campus	219 (73)

Total	**300**

**UCLA Status**	

Undergraduate	227(75.7)

Graduate Student	42(14.0)

Faculty	1(0.3)

Staff	15(5)

Other	15(5)

Total	**300**

185 (61.7%) were female and 115 (38.3%) were male. 227 (75.7%) were UCLA undergraduate students, 42(14.0%) were graduate students, 1(.3%) was a faculty member and 15 (5%) were classified as "other." 146 (48.7%) were White, non-Latino, 93 (31.0%) participants were Asian/Pacific Islander, 36 (12.0%) identified as Latino, 14 (4.7%) identified as Black, Non-Latino, 6 (2.0%) identified as Mixed race, 2 (.7%) individuals were Native American, and 3 (1.0%) individuals declined to specify. 81 (27%) reported that they had a known exposure to an individual with H1N1. 187 (62.3%) reported that they had had flu-like symptoms since the beginning of the previous season. Only 112 (37.3%), however, met the May 2010 CDC definition of influenza-like illness (fever AND cough and/or sore throat). Of those 112 individuals, only 72 people tested positive for H1N1 using a ≥ 1:40 dilution cut-off by HAI. 86 people (54.4%) tested positive for H1N1 and did not report experiencing symptoms meeting the May 2010 CDC definition of influenza-like illness. Among those who tested positive for Influenza A(H1N1) 2009, diarrhea and vomiting were the least commonly reported symptoms (18.4% and 10.8% respectively), whereas sore throat and stuffy nose were the most commonly reported symptoms (63.3% and 60.8% respectively). Symptomatic distributions of those individuals who tested positive for influenza A(H1N1) 2009 are shown in Table 2.

**Table 2:** Distribution of symptoms among those who tested positive for influenza A(H1N1) 2009

Symptom	No (%) with reported symptom
Chills	52 (32.9)

Cough	92 (58.2)

Diarrhea	29 (18.4)

Fever	75 (47.5)

Headache	83 (52.5)

Muscle Aches	58 (36.7)

Nausea	35 (22.2)

Runny Nose	93 (58.9)

Sore Throat	100 (63.3)

Stuffy Nose	96 (60.8)

Vomiting	17 (10.8)

Met May 2010 CDC definition of ILI	72 (45.6)

Reported Experiencing No Symptoms*	49 (31.0)

*17 of the 49 people (35.0%) who reported experiencing no symptoms also reported receipt of the 2009 H1N1 vaccine

**Sera were considered positive at an HI titre of 1:40 or greater

While 85 (28.3%) participants reported that they had received "any" flu shot, only 61 (20.3%) of the participants reported that they had received the H1N1 flu shot. 8 individuals (2.7%) reported that they had been previously diagnosed with H1N1 by a health professional.

Results of the hemagglutination inhibition assay are shown in Table 3. Demographic characteristics of participants both by vaccination and seropositivity status are reported in Table 4.

**Table 3:** Distribution of 2009 H1N1 hemagglutination inhibition assay results

H1N1 Lab Results		
< 10	80	26.7%

10	25	8.3%

20	14	4.7%

40	20	6.7%

80	44	14.7%

160	42	14.0%

320	25	8.3%

≤ 640	27	9.0%

No Results	21	7.0%

No Sample	2	0.7%

**Table 4:** Distribution of demographic characteristics by vaccination and seropositivity status

Characteristics	No (% of subgroup) vaccinated	No (% of subgroup) seropositive
**Gender**		

Male	28 (24.4)	66 (41.77)

Female	33 (17.8)	92 (58.23)

Total	**61**	**158**

**Ethnicity**		

Asian/Pacific Islander	23 (24.7)	58 (62)

Black, Non-Latino	0 (0)	9 (64)

Latino	5 (13.9)	20 (55)

Native American/Alaskan	1 (50)	1 (50)

White, Non-Latino	31 (21.2)	67 (45.9)

Mixed	1 (16.7)	2 (33.3)

Not Specified	0 (0)	1 (33)

Total	**61 (20.3)**	**158 (52.7)**

Even when using a very sensitive cut-off of ≥ 1:40 for the HAI results, 10 of 61 (16%) individuals who self-reported having specifically received the H1N1 vaccine did not test positive. Demographic and serologic characteristics of those who reported receipt of the 2009 H1N1 vaccine are shown in Table 5, whereas demographic and serologic characteristics of those who did not report receipt of the 2009 H1N1vaccine are shown in Table 6.

**Table 5:** Demographic and serologic characteristics among those who reported receipt of 2009 H1N1 vaccine

Characteristics	Number (% of subgroup) seropositive	Number (% of subgroup) seronegative
**Gender**		

Male	24 (85.7)	3 (10.7)

Female	26 (78.8)	7 (21.2)

Total	**50 (82.0)**	**10 (16.4)**

**Ethnicity**		

Asian/Pacific Islander	19 (82.6)	4 (17.4)

Black, Non-Latino	0 (0)	0 (0)

Latino	5 (100.0)	0 (0)

Native American/Alaskan	1 (100.0)	0 (0)

White, Non-Latino	25 (80.6)	5 (16.1)

Mixed	0 (0)	1 (100.0)

Not Specified	0 (0)	0 (0)

Total	**50 (82.0)**	**10**

*Table does not include those with no results or no sample from HAI, therefore some percentages might not sum to 100

**Table 6:** Demographic and serologic characteristics among those who did not report receipt of 2009 H1N1 vaccine

Characteristics	Number (% of subgroup) seropositive	Number (% of subgroup) seronegative
**Gender**		

Male	42 (48.3)	38 (43.7)

Female	66 (43.4)	71 (46.7)

Total	**108 (45.2)**	**109 (45.6)**

**Ethnicity**		

Asian/Pacific Islander	39 (55.7)	25 (35.7)

Black, Non-Latino	9 (64.3)	4 (28.6)

Latino	15 (48.4)	12 (38.7)

Native American/Alaskan	0 (0)	1 (100.0)

White, Non-Latino	42 (36.5)	63 (54.8)

Mixed	2 (40.0)	3 (60.0)

Not Specified	1 (33.3)	1 (33.3)

Total	**108 (45.2)**	**109**

*Table does not include those with no results or no sample from HAI, therefore some percentages might not sum to 100

**Table includes those who were unsure of receipt of the 2009 H1N1 vaccine

## Conclusions

Overall, 52.7% of the total study population tested positive for influenza A(H1N1) 2009. Because of the convenience sampling design, it is unclear if this is representative of the UCLA population in general or if the study population was comprised of highly motivated participants. Even if it was representative of UCLA, it is unclear if the results would be generalizable to all universities in general or even just to all large public universities. 54.4% of those who tested positive for influenza A(H1N1) 2009 in this study population, using the ≥ 1:40 dilution cut-off on the hemagglutination inhibition assay, did not meet the May 2010 CDC definition for influenza-like illness. Furthermore, 49 (31%) people who tested positive did not report experiencing any flu-like symptoms at all. Of those individuals, 32 (65.3%) also did not report receiving the 2009 H1N1 vaccine, therefore we believe them to have been truly asymptomatic cases based on self reporting of symptoms. Although the potential existed for inaccurate reporting of symptoms and vaccination given the population of college students and the long time frame about which they were asked to recall, the questionnaire was specifically designed to minimize such bias. This suggests that either a large number of cases of influenza A(H1N1) 2009 are asymptomatic or subclinical, presenting with either absent or mild symptoms that were not readily observable; the 1:40 dilution cut-off on the hemagglutination inhibition assay had low specificity or there was poor specificity on the May 2010 CDC case definition. Since previous serosurveys did indicate high levels of asymptomatic or subclinical infection, we believe that many individuals in our population were asymptomatically or subclinically infected as well [5]. If nearly half of those infected do not mount any symptoms of infection or symptoms so mild as to avoid detection by the individual this represents a special population that may have both positive and negative impacts on preparedness efforts. This potentially large asymptomatic or subclinical population would be less likely to engage in isolation or social distancing measures that have been proven to be effective given that they are unaware of their infection and therefore of their ability to transmit the virus [24]. Additional research regarding such ability of these asymptomatic individuals to transmit the virus needs to be done [25,26]. Conversely, if these asymptomatic or subclinically infected individuals simultaneously mount a protective immune response to the infection, then fewer people will need to be vaccinated in order to generate herd immunity to the virus. However, the possibility must be considered that a 1:40 dilution, which is the widely used field standard for serosurveys, does not actually indicate the presence of lasting protective immunity. Therefore, this seroprevalance data cannot independently be used to generate conclusions about herd immunity and must be used in conjunction with information on the R_0 _of this virus. Until these critical issues are resolved it is important that individuals follow the vaccination guidelines set forth by national and international health agencies.

Because of the location and timing of the study on the UCLA campus, any undergraduate or graduate student would have been eligible to receive free H1N1 vaccine through the University health service, yet only 20.3% of the total study population reported that they had chosen to receive the vaccine. We contacted the University health service and found that the total number of H1N1 vaccines given out by their office to students at UCLA during the period from which they received the vaccine and began to make it available (19 October 2009 until 3 March 2010) was 1802. Using the Fall 2009 enrollment information at UCLA as a denominator, we calculated that the total prevalence of vaccination on campus was 1802 out of 38556 total students, or 4.67%. It is our thought that the discrepancy in vaccination prevalence between our study and the total UCLA population was partially due to the method of vaccine administration. The only vaccine available on the UCLA campus was injection and, as we were asking patients to give a blood sample in our study, we will assume that our study population represented a highly motivated and non-needle averse group of individuals. Large differences in vaccination coverage were also seen by self-reported race/ethnicity. None of the individuals who self-identified as black, reported that they had been vaccinated with the H1N1 vaccine. However, this is consistent with other previously published studies indicating difficulty in reaching the African-American population in H1N1 vaccination campaigns [27,28]. Lower reported vaccination was also seen in the Latino participants as compared to the overall study population. While females appear to be more likely to have been infected with influenza A(H1N1) 2009 (58.23% vs. 41.77%), males appear to be slightly more likely to have reported being vaccinated against influenza A(H1N1) 2009 (24.4% vs. 17.8%). 16% of those who reported vaccination did not test positive through the Hemagglutination inhibition assay using a ≥ 1:40 dilution cut-off. It is possible these negative results occurred because of problems with the shipping or storage of the blood specimens, or because of problems with the hemagglutination inhibition assay. However, since the positive and negative controls that were run with the assays all turned out appropriately, laboratory error is not the most likely explanation. Since all individuals who were recruited into the study were young and healthy, it is unlikely that these results can be explained because of anergic participants. One possibility is that this was a result of recall bias and that participants were unable to appropriately recall if they had received the seasonal influenza vaccine or the H1N1 vaccine. It is also possible that some individuals were vaccinated with product that was sub-standard, or it is possible that for some individuals, one dose of vaccine was not sufficient to stimulate protective immunity against influenza A(H1N1) 2009. Early in the 2009 epidemic, the Food and Drug Administration's designation was that all eligible individuals would need to receive two doses of the H1N1 vaccine [29]. However, clinical trials showed that one dose of vaccine was highly immunogenic in adults [30]. Shortly thereafter, they reassessed their position, and stated that one dose of vaccine against influenza A(H1N1) 2009 virus would be sufficient for individuals 10 years of age and older and only those children 6 months through 9 years of age would need two doses [31]. Unfortunately, since influenza A(H1N1) 2009 is now a component of the seasonal vaccine, it is not possible to assess the immunogenicity of the vaccine using the same methodology.

## Competing interests

The authors declare that they have no competing interests.

## Authors' contributions

SCS conceived of and executed the study and drafted the manuscript. KAO performed the statistical analysis and helped to draft the manuscript. KIS participated in the design and coordination of the study. All authors read and approved the final manuscript.

## Authors' information

Dr Shafir recently completed her PhD in infectious disease epidemiology at the UCLA School of Public Health and is working there as an assistant professor in the Department of Epidemiology. She and Dr. Shoaf both work at the UCLA Center for Public Health and Disasters and work on Disaster Preparedness. Ms. O'Keefe is a doctoral student in the UCLA Department of Epidemiology with a strong interest in infectious disease epidemiology.

## Pre-publication history

The pre-publication history for this paper can be accessed here:

http://www.biomedcentral.com/1471-2458/11/922/prepub
